# Alteration of the serum microbiome composition in cirrhotic patients with ascites

**DOI:** 10.1038/srep25001

**Published:** 2016-04-26

**Authors:** Alba Santiago, Marta Pozuelo, Maria Poca, Cristina Gely, Juan Camilo Nieto, Xavier Torras, Eva Román, David Campos, Guillaume Sarrabayrouse, Silvia Vidal, Edilmar Alvarado-Tapias, Francisco Guarner, German Soriano, Chaysavanh Manichanh, Carlos Guarner

**Affiliations:** 1 Department of Gastroenterology, Vall d’Hebron Research Institute, Passeig Vall d’Hebron 119-129, Barcelona 08035, Spain; 2 Department of Gastroenterology, Hospital de la Santa Creu i Sant Pau, Barcelona, Spain; 3 Centro de Investigaciòn Biomédica en Red en el Área temática de Enfermedades Hepáticas y Digestivas, CIBERehd, Instituto de Salud Carlos III, Madrid, Spain; 4 Department of Immunology, Institut de Recerca-IIB Sant Pau, Hospital de la Santa Creu i Sant Pau, Barcelona, Spain; 5 Escola Universitària d’Infermeria EUI-Sant Pau, Hospital de la Santa Creu i Sant Pau, Barcelona, Spain

## Abstract

The progression of cirrhosis is associated with alterations in the composition of the gut microbiome. To assess microbial translocation, we compared the serum microbial composition of patients with and without ascites and characterized the ascitic fluid microbiome using 16S rDNA high-throughput sequencing data. A complex and specific microbial community was detected in the serum and ascitic fluid of patients with cirrhosis but barely detectable in the serum of healthy controls. The serum microbiome of patients with ascites presented higher levels of lipopolysaccharide binding protein, a marker of microbial translocation, associated with higher diversity and relative abundance of Clostridiales and an unknown genus belonging to the Cyanobacteria phylum compared to patients without ascites. The composition of the fecal microbiome was also more altered in patients with than without ascites, confirming previous studies on fecal microbiome. We propose that alteration of the serum and fecal microbiome composition be considered indicators of cirrhosis progression.

Liver cirrhosis is a major cause of global health loss. In this regard, its incidence increased from 676,000 patients in 2008 to over 1 million in 2010^1^. It is the final phase of chronic liver disease, in which inflammation is associated with dying hepatic cells and fibrosis, leading to poor liver function and portal hypertension. Alterations in the gut microbiota, which represents the collective microbial cells present in the digestive tract, or its products, are linked to the progression of liver disease and the complications of cirrhosis^2^. Over the last decade, advances in molecular techniques and bioinformatics, as well as the exponential decrease in the cost of sequencing, have allowed comprehensive characterization of the composition and function of the gut microbial community. Using these techniques, recent studies on the gut microbiome have demonstrated an alteration of the composition of the stool microbial community in cirrhotic patients compared to healthy controls^3^,^4^. Furthermore, this level of alteration appears to be positively correlated with the severity of the disease^5^.

More specifically, bacterial translocation has been suspected to play an important role in the pathogenesis and complications of cirrhosis. By administering green fluorescent protein (GFP)-labeled *Escherichia coli* orally to cirrhotic rats, Teltschik *et al*.^6^ revealed the presence of bacteria not only in the intestinal lumen but also in mesenteric lymph nodes (MLNs) and ascites. We also recently described that rat MLNs harbor a high microbial diversity^7^. However, very little is known about the microbiome of extra-intestinal sites such as the systemic circulation and ascitic fluid in patients with cirrhosis.

This study sought to: (a) characterize the microbiome of serum and fecal samples of patients with cirrhosis and compare them with those of healthy controls; (b) define the serum microbiome associated with severity of liver disease; and (c) identify the microbiome of ascitic fluid.

## Results

### Enrollment process

A total of 60 outpatients with cirrhosis were evaluated. Thirty-three were excluded due to treatment with non-absorbable disaccharides and/or antibiotics (*n* = 11), current alcohol intake (*n* = 7), hepatocellular carcinoma (*n* = 5), spontaneous bacterial peritonitis (*n* = 1), other infections or suspicion of infection (*n* = 3), severe comorbidities (*n* = 4), or because they were unwilling to participate in the study (*n* = 2). Therefore, a total of 27 patients were included—13 with ascites and 14 without ascites. Seventeen healthy controls were included for stool (*n* = 17) and serum (*n* = 7) microbiome analysis.

### Patient characteristics

The characteristics of patients are shown in Supplementary Table 1. The main differences between the two groups of patients consisted, as expected, of a more advanced liver insufficiency as determined by the Child-Pugh score and a higher incidence of previous ascites in patients with than in those without ascites. When analyzing other factors that could influence the microbiome composition, we did not find statistical differences between the two groups regarding age, body mass index or etiology of cirrhosis. Patients without ascites showed a trend towards a lower prevalence of diabetes than those with ascites and they were more frequently receiving treatment with beta-blockers or proton pump inhibitors. These differences, however, did not reach statistical significance.

No patient in either group presented symptoms, signs at physical examination or analytical data suggesting infection. Microbial cultures were negative, and neutrophil count was <250/mm^3^ in all ascitic fluid samples. Therefore, all patients with ascites were considered to have a non-infected ascitic fluid.

### Microbiome in stool

**Figure 1:**
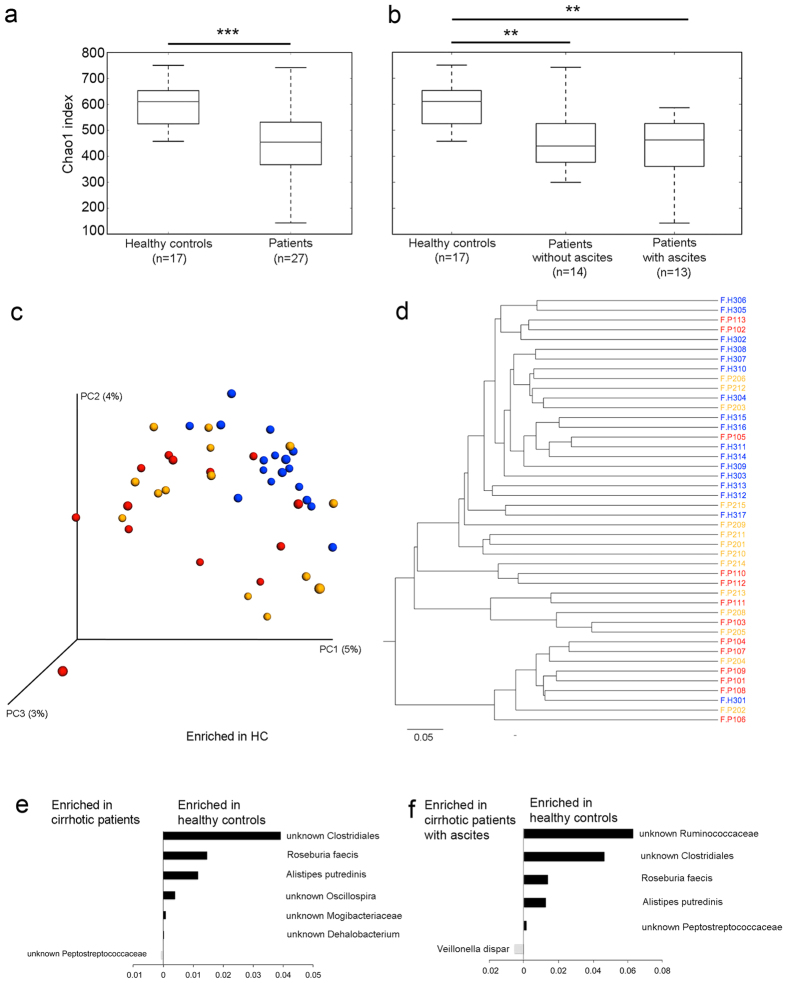
Fecal microbiome of cirrhotic patients and healthy controls. (**a,b**) Healthy controls presented higher microbial diversity compared to all cirrhotic patients (**a**) and to patients with and patients without ascitic fluid (**b**) as assessed by the Chao1 index. The two groups of patients with and without ascites were not significantly different. (**c,d**) Unweighted UniFrac PcoA (**c**) and weighted UniFrac UPGMA (**d**) clustering analysis. Blue: healthy controls; orange: patients without ascites; and red: patients with ascites. (**e,f**) Relative abundance of microbes differentially present at the species level between healthy controls and all cirrhotic patients (**e**) and between healthy controls and cirrhotic patients with ascites (**f**) (Kruskal-Wallis; FDR < 0.05). Analyses were performed on 16 S rRNA V4 region data, obtained from stool samples, rarefied to a depth of 19,930 reads per sample. Healthy controls (*n* = 17); patients (*n* = 27); patients with ascites (*n* = 13); patients without ascites (*n* = 14); ***P = 0.001; **P = 0.003.

The stool microbiome of 27 patients with cirrhosis was compared to that of 17 healthy controls. Alpha-diversity analysis showed that the fecal microbial community of healthy controls presented a higher diversity than that of patients with cirrhosis (Fig. 1a). However, the diversity was similar in patients with or without ascites (Fig. 1b). Together, these results suggest that a loss of microbial diversity in fecal samples is associated with cirrhosis without ascites, but the progression to ascites is not associated with a further loss of diversity. Clustering analysis using PCoA and UPGMA methods based on UniFrac metrics showed that the stool microbiome of cirrhotic patients clustered separately from that of healthy controls (Fig. 1c,d). At the taxonomic level, patients with cirrhosis were depleted of six species (FDR < 0.05; Kruskal-Wallis test): unknown Clostridiales, *Roseburia faecis, Alistipes putredinis*, unknown *Oscillospira*, unknown Mogibacteriaceae, and unknown *Dehalobacterium*, but were enriched in an unknown Peptostreptococcaceae compared to healthy controls (FDR < 0.05; Kruskal-Wallis test; Fig. 1e). Proteobacteria, at the phylum level, were more abundant in cirrhotic patients than in healthy controls but the difference did not reach significance (FDR = 0.42; Kruskal-Wallis test). All together, these results confirm previous findings that the microbiome composition of cirrhotic patients is altered^4^.

Cirrhosis can progress to ascites, which is defined as the accumulation of fluid in the peritoneal cavity. Interestingly, when we analyzed the stool microbiome of patients with ascites and those without ascites separately, only the former displayed a significant dysbiosis at the species level, with depletion of unknown Ruminococcaceae, Clostridiales and Peptostroptococcaceae, *Roseburia faecis* and *Alistipes putredinis* and with an enrichment of *Veillonella dispar* compared to healthy controls (FDR < 0.05; Kruskal-Wallis test; Fig. 1f). For several of these species, such as *Roseburia faecis, Alistipes putredinis* and *Veillonella dispar,* our findings are in line with those of Qin *et al*.^4^ and further support the notion that the progression of the disease is associated with a greater dysbiosis, as reported by Bajaj *et al*.^5^. Patients without ascites presented only a trend towards lower relative abundance of unknown Mogibacteriaceae and *Alistipes* (FDR = 0.053; Kruskal-Wallis test) compared to healthy controls.

### Microbiome in fluids

Standard diagnostic microbiological analysis revealed that the serum and ascitic fluid samples were negative for bacterial growth. We analyzed the microbiome serum from 7 healthy controls and from the 27 patients and ascitic fluid from 11 patients. Analysis of the 16S rRNA gene of such low-biomass samples may generate contamination at various steps of the process. Therefore, we applied strict protocols for sample collection, DNA extraction, and PCR amplification. For sample collection, we used gloves and proceeded in sterile conditions. For DNA extraction, we used chemicals such as DNA terminator (Biotools, B & M Labs, Spain) to degrade any trace of contaminant DNA in laboratory equipment, and we added negative controls (blanks) during extraction. During PCR amplification, we used UV to clean consumables and H2O and also added PCR blanks.

The amplicons were analyzed in an electrophoretic gel and their presence was indicated by a DNA band at about 400 bp (Supplementary Fig. 1). No DNA band was observed for four control serum samples out of seven, one serum sample from patient with ascites and one ascitic fluid sample, or for the negative controls added during the extraction (NEG1 and NEG2) and PCR (NEG3) procedures. The PCR amplifications of serum and ascitic fluid samples provided a gradient of intensity in the DNA bands, as analyzed in the electrophoretic gel (Supplementary Fig. 1), in the following order: healthy control serum < cirrhotic patients without ascites < cirrhotic patients with ascites < ascitic fluid, thereby also suggesting a gradient in the microbial load. To remove potential false positive OTUs during sequence analysis, we subtracted sequences with abundant taxa generated in the blanks from the serum and ascitic fluid samples and applied a more restricted filter to the data obtained from samples in order to remove taxa with a low abundance, as specified in the method section. The contamination present in the negative controls was identified as being mostly Proteobacteria (69%) at the phylum level and Pseudomonas (30%), Halomonas (18%) and unknown (12%) at the genus level. After this filtering step and at a rarefaction of 1000 sequences per sample, we obtained sequence data for 24 out of 27 serum samples from patients and for eight out of 11 ascitic fluid samples and no sequence data were recovered from healthy controls. Supplementary Fig. 2 shows the taxonomic profiling of the three sample types at the phylum level before and after the sequence-filtering step.

**Figure 2:**
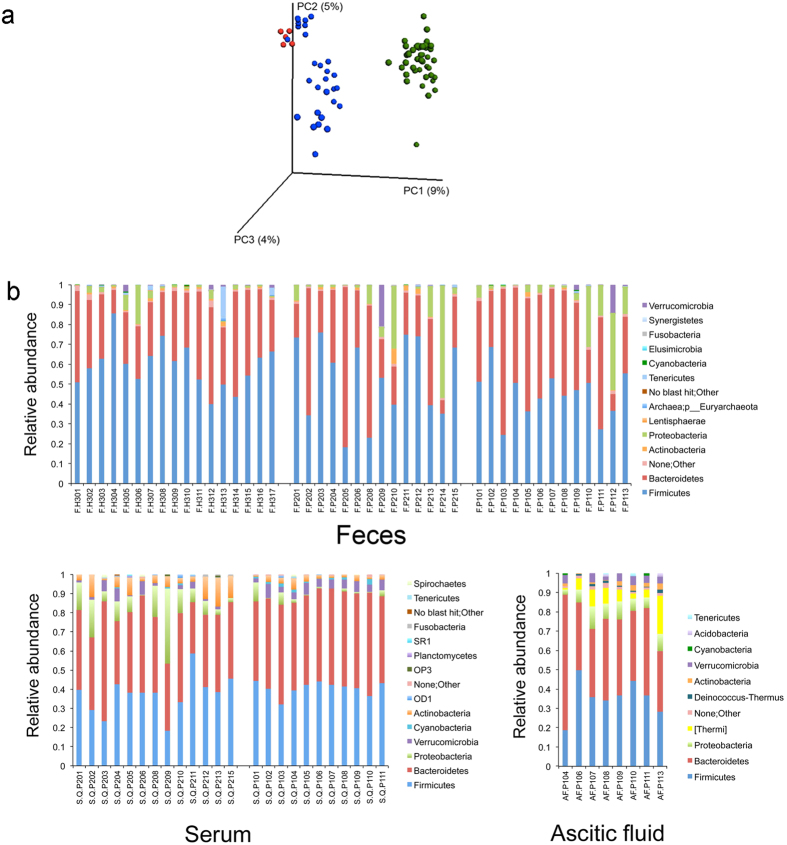
Fecal, serum and ascitic fluid microbiome. (**a**) Clustering of samples using unweighted UniFrac PcoA representation. (**b**) Taxonomic composition at the phylum level of the three sample types: Feces, serum, and ascitic fluid. Analyses were performed on 16 S rRNA V4 region data, rarefied to a depth of 19,930 reads for stool and 1,000 reads for serum and ascitic fluid samples. Green: stool; blue: serum; red: ascitic fluid. F.H = Feces of healthy controls; F.P = Feces of patients with cirrhosis; S.Q.P = Serum of patients; AF.P = ascitic fluid of patients. 201 to 215 = patients without ascites; 101 to 113 = patients with ascites.

Beta-diversity analysis, which studies the variation in composition between samples, showed a similar microbial composition between serum and ascitic fluid samples. However, the microbial community differed greatly between these two sample types and the stools (Fig. 2a), although 89% and 86% of the serum and ascitic fluid microbiome was shared with the stool microbiome at the genus level (Supplementary Fig. 3). Euryarchaeota (phylum level) was detected only in stool samples and Thermi and Deinococcus-Thermus were detected only in ascitic fluid (Fig. 2b). Firmicutes and Bacteroidetes were the two most dominant phyla in the three sample types.

**Figure 3:**
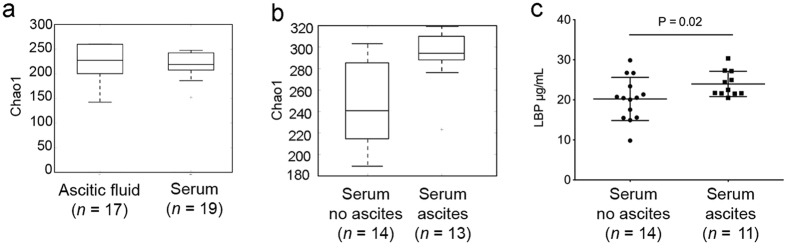
Microbial of extra-intestinal sites and marker of translocation. (**a**) Alpha-diversity of the microbial fluid samples as assessed by Chao1 index of diversity. Ascitic fluid (*n* = 11); Serum of patients with cirrhosis (*n* = 19; instead of 27 due to rarefaction depth with ascitic fluid samples). (**b**) Higher alpha-diversity of serum microbiome of cirrhotic patients with ascites compared to that of patients without (*P* < 0.05). Analyses were performed on 16 S rRNA V4 region data, rarefied to a depth of 1,000 reads per sample. (**c**) Lipopolysaccharide binding protein (LBP) levels as assessed by specific ELISA; serum of patients with ascites (*n* = 11 available samples).

From serum and ascitic fluid, we detected six and eight groups of microbes at the phylum level, 26 and 28 groups at the family level, and 36 and 38 groups at the genus level, respectively. At the phylum level, Firmicutes (41%), Bacteroidetes (37%) and Proteobacteria (14%) accounted for 92% of the sequence data of the serum microbiome, whereas in ascitic fluid Firmicutes (46%), Bacteroidetes (27%), Thermi (10%) and Proteobacteria (8%) accounted for 92%. Serum and ascitic fluid were similar in terms of diversity and richness, as assessed by an abundance-based richness estimator (Chao1) (Fig. 3a). However, serum specimens of patients with ascitic fluid presented a more diverse microbiome (*P* = 0.008) than those of patients without (Fig. 3b), and a significantly higher concentration of lipopolysaccharide binding protein (LBP) (*P* = 0.02, Mann Whitney test), a marker of microbial translocation (Fig. 3c). This observation could be explained by patients with ascites, who are expected to have a greater deterioration of the intestinal barrier integrity, also having a higher degree of microbial translocation than those without ascites, thus leading to a higher microbial diversity in serum.

**Figure 4:**
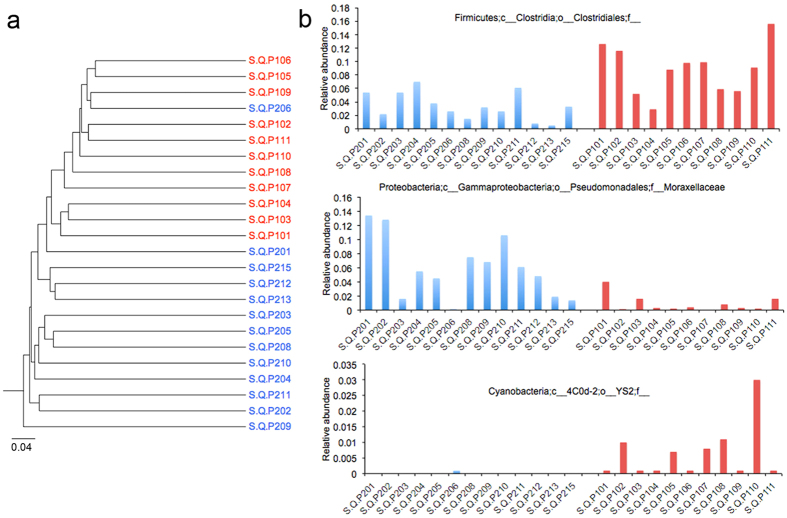
Serum microbiome of patients with and without ascites. (**a**) UPGMA clustering based on unweighted UniFrac metric of serum samples of cirrhotic patients with and without ascites. (**b**) Relative abundance of microbes or groups of microbes significantly different between serum microbiome of cirrhotic patients with and without ascites. Analyses were performed on 16S rRNA V4 region data, rarefied to a depth of 1,000 reads per sample.

Furthermore, using an UPGMA clustering method of the serum microbiome based on an unweighted UniFrac metric, the microbiome of patients with and without ascites clustered separately (Fig. 4a). This result suggests that a specific serum microbiome is linked to the presence of ascites.

Taxonomic comparison showed that an unknown group of microbes at the family level, belonging to the Clostridiales order, displayed a higher relative abundance in serum of patients with ascites (FDR = 0.03; Kruskal-Wallis test) and another group, Moraxellaceae, showed a lower relative abundance in patients with ascites compared to those without (Fig. 4b). Interestingly, this group of bacteria was also found in ascitic fluid samples (Supplementary Fig. 4), thereby supporting the notion of translocation from serum to ascitic fluid. At the genus and species level, an unknown genus related to Cyanobacteria (FDR = 0.002) was found in higher relative abundance in patients with ascites compared to those without.

### Microbial translocation

In order to study whether the presence of bacterial DNA in ascitic fluid and blood derived from the gastrointestinal tract, we counted the taxa common to stool and serum, stool and ascitic fluid, and serum and ascitic fluid. For this purpose, we first counted the number of taxa in each sample type, finding an average of 397 (SD = 94), 283 (SD = 76) and 97 (SD = 25) taxa in stool, serum and ascitic fluid, respectively. By comparing the taxa between samples, we detected on average 37 taxa common to both stool and serum, 20 to serum and ascitic fluid, and three to ascitic fluid and stool (Supplementary Fig. 5). These results indicate that the three sites share few common microbial taxa and therefore suggest that the microbial taxa present in the serum, but not detected in stool, could either take root in extra-intestinal sites such as the lung or the vagina for women or were in too low abundance in the stool to be detectable but when they reached the serum, a more appropriate environment for their growth, they became detectable.

## Discussion

This is the first study to validate the presence of polymicrobial DNA in both the serum and ascitic fluid of patients with cirrhosis using high-throughput sequencing techniques. Our findings showed that the microbial community in serum and ascitic fluid, although showing more than 80% similarity with that of the stool microbiome at the genus level, is specific and complex at the taxa level. Previous studies using a variety of techniques, mainly conventional PCR, reported the presence of bacterial DNA in ascitic fluid and/or blood only in up to 30–60% of these patients^8^,^9^,^10^,^11^. Moreover, most of the DNA detected in these studies was monomicrobial, identified as being *Escherichia coli* or *Staphylococcus aureus*^8^,^9^,^10^,^11^. A recent study has reported the characterization of the microbial composition of the ascitic fluid of cirrhotic patients^12^. However, the authors amplified the 16 S gene from only one individual out of seven and this individual was positive for *Escherichia coli* in culture. Using shotgun-sequencing technique on two pools of ascitic fluid obtained from three patients, they were able to identify only 0.1% of bacterial DNA, for which the majority was identified as being *Escherichia*. However, according to our findings, *Escherichia* belonging to the Proteobacteria phylum could also be found in the extraction and PCR blanks. We therefore recommend that future studies on samples with a low biomass include several blanks and minimize the amount of Taq polymerase used during the PCR amplification, since it may contain contaminant DNA. The detection of polymicrobial DNA in the serum and ascitic fluid observed in the present study is in line with our previous findings in rats, showing a high microbial diversity in MLNs of a model of CCl4-induced liver injury, as well as in those of control rats^7^.

We were unable to analyze the serum microbiome of the seven healthy controls at a sufficient rarefaction depth compared to all other samples. Indeed, the presence of DNA bands in the electrophoretic gel after serum amplification could be due to the presence of human DNA combined with contaminant DNA during extraction and amplification, thus impeding analysis of the microbiome of these samples after filtering out the contaminant sequences. As the same method of sample collection and processing was used for patients with cirrhosis, this finding supports that the detection of bacterial DNA in patients with cirrhosis was not caused by contamination. This observation suggests that healthy individuals harbor a very low or undetectable microbial load in blood, which is in agreement with a recent study demonstrating the presence of a gut-vascular barrier that controls the systemic dissemination of bacteria in healthy individuals but not in patients with celiac disease and liver damage^13^. In cirrhotic patients, the similarity of the microbiome composition between serum and ascitic fluid compared to stool samples could be due, in part, to the body site selecting only microorganisms capable of growing in a liquid and relatively aerobic environment. The differences found in diversity (Chao1 index) and in composition and structure of the serum microbiome between patients with and without ascites are alterations that are associated with cirrhosis progression, thereby validating the assumption of previous studies^14^.

The decrease in stool microbial diversity and the depletion of several commensal groups of bacteria (unknown Ruminococcaceae, Clostridiales and Peptostroptococcaceae, *Roseburia faecis* and *Alistipes putredinis*) in patients with cirrhosis is also in agreement with the findings of previous studies^3^,^4^. However, in contrast to other authors^5^, we did not observe a significant increase in potential pathogenic bacteria such as Enterobacteria, but only a trend towards an increase in Proteobacteria or Streptococcaceae. This observation could be explained by a smaller sample size and the fact that the patients in our study presented a relatively preserved liver function, as reflected by the low Child-Pugh and MELD scores, in comparison with other studies that included groups with more advanced liver failure.

Our study presents several limitations such as a small sample size, DNA contamination that may remain after sequence curating (despite the multiple precautions to avoid this as mentioned above), and confounding factors. To reduce possible confounding factors, we excluded patients with recent alcohol intake and those treated with antibiotics or non-absorbable disaccharides. We did not find statistically significant differences between patients with and without ascites in other possible confounding factors, such as diabetes and the use of beta-blockers or proton-pump inhibitors. However, we cannot exclude that the non-significant differences observed in these parameters could have influenced the results reported here.

Despite these limitations, we conclude that serum and ascitic fluid of patients with cirrhosis contain a complex and specific microbial community and that our method of low-biomass analysis could be applied to other conditions of gut-vascular barrier failure^13^. We propose that alteration of the serum and fecal microbiome composition be considered indicators of cirrhosis progression.

## Methods

### Ethical statement

The study included consecutive outpatients with cirrhosis treated at the *Hospital de la Santa Creu i Sant Pau*, a tertiary care hospital in Barcelona, Spain. The methods conformed to the Declaration of Helsinki and Guidelines for Good Clinical Practice in Clinical Trials and were carried out in accordance with the Clinical Research Ethics Committee of the *Hospital de la Santa Creu i Sant Pau*. All experimental protocols were approved by the same Ethics Committee. All patients received information concerning their participation in the study and gave written informed consent.

### Patient information

Cirrhosis was diagnosed by clinical, analytical, and ultrasonographic findings or by liver biopsy. Exclusion criteria were the following: hospitalization in the previous month due to decompensation of cirrhosis; hepatocellular carcinoma or other neoplasia; alcohol intake in the previous 3 months; current infection or overt hepatic encephalopathy; marked symptomatic comorbidities (cardiac, pulmonary, renal, untreated active depression); treatment with antibiotics or non-absorbable disaccharides in the previous 3 months; and life expectancy of less than 6 months. Patients were carefully evaluated to exclude active infection when joining the study. Patients were classified into two groups, namely those with ascites and those without. The former group consisted of stable patients with refractory ascites attending the day hospital for regular therapeutic paracentesis. A group of age- and gender-matched healthy controls was included to compare their stool and blood microbiome composition with that of patients with cirrhosis.

### Sample collection

Fecal samples were collected by the patients or controls as previously described^15^. Blood and ascitic fluid samples were collected in sterile conditions by peripheral vein puncture and during therapeutic paracentesis, respectively. For patients with cirrhosis, we performed routine blood analysis to assess the degree of liver failure, renal function, blood white cell count, and ascitic fluid neutrophil count to rule out ascitic fluid infection (spontaneous bacterial peritonitis). Samples of blood and ascitic fluid were cultured in blood culture bottles (BactAlert^®^) to assess for microbial growth. Additional samples of blood and ascitic fluid were collected in in SST™ Tubes (BD Vacutainer®) tubes and 15 ml centrifuge tubes respectively, and frozen at −80 °C until DNA analysis.

### Lipopolysaccharide binding protein levels

Serum was tested for lipopolysaccharide binding protein (LBP) concentration to assess exposure to bacteria and their endotoxins as an index of bacterial translocation^16^,^17^, using specific ELISA (Biometec GmbH, Greifswald, Germany) according to the manufacturer’s instructions. LBP was quantified with standard curves provided by the corresponding ELISA kit. The detection limit was 5 ng/mL.

### DNA extraction, PCR amplification, and sequencing

We analyzed the microbiome of samples from healthy controls (stool, *n* = 17; serum, *n* = 7) and cirrhotic patients (stool, *n* = 27; serum, *n* = 27; ascitic fluid, *n* = 11). In order to identify possible contamination in low-biomass samples and subtract the sequences of the potentially contaminated DNA generated during the extraction and PCR amplification, we introduced negative controls (blanks) during these two technical steps.

A frozen aliquot of fecal sample (250 mg) from each individual (*n* = 44) was subjected to genomic DNA extraction using a previously described method, referred to here as the “Godon” method^15^,^18^. Each sample was suspended in 250 μl of guanidine thiocyanate, 0.1 M Tris (pH 7.5), 40 μl of 10% N-lauroyl sarcosine, and 500 μl 5% N-lauroyl sarcosine. DNA was extracted by mechanical disruption of the microbial cells with beads. RNA was removed by the addition of 2 μl of a 10-mg/ml solution of RNAase, and nucleic acids were recovered from clear lysates by alcohol precipitation. Twenty-seven and seven serum samples were collected from patients and healthy controls, respectively, and subjected to genomic DNA extraction using beads to disrupt the microbial cells followed by the QIAamp® DNA Blood Midi Kit (Qiagen, Madrid, Spain), following the manufacturer’s protocol. We obtained 11 ascitic fluid samples (4 ml) from 13 patients. Microbial DNA was extracted using a modified “Godon” protocol. In this regard, after a 10-min centrifuge at 14000 rpm, the pellet was subjected to the same procedure as the fecal samples. However, the final resuspension of the nucleic acids was carried out with 30 μl of a Tris-EDTA buffer solution.

An equivalent of 1 mg of each sample was used for DNA quantification using a NanoDrop ND-1000 Spectrophotometer (Nucliber). DNA integrity was examined by micro-capillary electrophoresis using an Agilent 2100 Bioanalyzer with the DNA 12,000 kit, which resolves the distribution of double-stranded DNA fragments up to 17,000 bp in length.

For profiling microbiome composition, the hyper-variable region (V4) of the bacterial and archaeal 16 S rRNA gene was amplified by PCR. On the basis of our analysis done with Primer Prospector software, the V4 primer pairs used in this study were expected to amplify almost 100% of the bacterial and archaeal domains. The 5′ ends of the forward (V4F_515_19: 5′-GTGCCAGCMGCCGCGGTAA-3′) and reverse (V4R_806_20: 5′-GGACTACHVGGGTWTCTAAT-3′) primers targeting the 16S gene were tagged with specific sequences as follows: 5′-{AATGATACGGCGACCACCGAGATCTACACTATGGTAATTGT}^3^,^15^,^18^ {GTGCCAGCMGCCGCGGTAA}-3′ and 5′-{CAAGCAGAAGACGGCATACGAGAT} {Golay barcode} {AGTCAGTCAGCC} {GGACTACHVGGGTWTCTAAT}-3′. Multiplex identifiers, known as Golay codes, had 12 bases and were specified downstream of the reverse primer sequence (V4R_806_20)^19^,^20^.

Standard PCR using 0.75 units of Taq polymerase (Roche) and 20 pmol/μL of the forward and reverse primers was run in a Mastercycler gradient (Eppendorf) at 94 °C for 3 min, followed by 35 cycles of 94 °C for 45 sec, 56 °C for 60 sec, 72 °C for 90 sec, and a final cycle of 72 °C for 10 min. Amplicons were first purified using the QIAquick PCR Purification Kit (Qiagen, Barcelona, Spain), quantified using a NanoDrop ND-1000 Spectrophotometer (Nucliber) and using an Agilent 2100 Bioanalyzer with the DNA 1000 kit, and then pooled in equal concentration. The pooled amplicons (2 nM) were then subjected to sequencing using Illumina MiSeq technology at the technical support unit of the Autonomous University of Barcelona (UAB, Spain), following standard Illumina platform protocols.

### Sequence analysis

Sequences obtained from stool, ascitic fluid, and serum, together with negative controls from the extraction and PCR methods, were analyzed with QIIME 1.8.0^21^ using an in-house script. Raw sequences of low quality were filtered out with a minimum acceptable Phred score of 20. A demultiplexing step was performed to assign back each read to its corresponding sample and to remove barcodes. A total of 3,393,253 high quality sequences were finally recovered (2,910,686 for feces and 482,567 for serum and asctic fluid samples). UCLUST algorithm based on 97% of similarity was used to cluster similar sequences into Operational Taxonomic Units (OTUs) or taxa. Representative sequences of each OTU were aligned using PyNAST against Greengenes template alignment (gg_13_8). Chimeric sequences were then identified and removed with ChimeraSlayer. Finally, a taxonomical assignment for each OTU was performed with the basic local alignment search tool (BLAST) and the combination of two microbial databases (Greengenes and PATRIC). A phylogenetic tree was obtained with the FastTree program. The general OTU table was split into various tables in order to individually analyze feces, serum, and ascitic fluid samples.

In order to avoid false positive OTUs in stool samples, we eliminated those that did not represent at least 0.2% of the sequences. For samples with a low biomass, such as serum and ascitic fluid, we removed the OTUs that did not account for at least 0.2% of the sequences in at least 3 samples. Moreover, OTUs detected in negative controls were also removed for downstream analyses. Unknown bacteria assigned by BLAST against Greengenes and PATRIC databases were additionally checked against the NCBI database, and OTUs identified as from human origin were removed from the dataset. The final total, mean, minimum and maximum number of sequences per sample type were computed, and OTU tables were rarefied at several rarefaction depths (Supplementary Table 2).

### Statistical analyses

The characteristics of patients with and without ascites were compared using Fisher’s exact test for categorical variables and Mann-Whitney test for quantitative variables. For sequence analysis, pairwise comparisons were performed using OTU tables generated from each sample type. Samples that contained fewer reads than the rarefaction depth were removed for the alpha and beta diversity analyses. The Shapiro-Wilk test was used to check normality of the data, and pairwise comparisons were made between the study groups with the non-parametric test Kruskal-Wallis one-way analysis of variance, which compares means between groups. False discovery rate (FDR) corrected p-values were taken into account to consider significant results. Richness provided by alpha diversity was computed with Chao1 index. Sample clustering was performed using UPGMA and PCoA methods based on UniFrac metrics.

## Additional Information

**Accession Code**: The 16S rRNA gene sequences have been deposited in the NCBI-SRA database under the accession BioProject ID: SUB1268941 (Temporary Submission ID).

**How to cite this article**: Santiago, A. *et al*. Alteration of the serum microbiome composition in cirrhotic patients with ascites. *Sci. Rep.* **6**, 25001; doi: 10.1038/srep25001 (2016).

## Supplementary Material

Supplementary Information
