# Supplementary material for: Alteration of the serum microbiome composition in cirrhotic patients with ascites
Source: Sci Rep. 2016 Apr 26;6:25001. doi: 10.1038/srep25001 (PMC4845009; doi:10.1038/srep25001)
Supplement: Supplementary Information [file srep25001-s1.doc]

**Alteration of the serum microbiome composition in cirrhotic patients with ascites**

**Authors**: Alba Santiago^1^, Marta Pozuelo^1^, Maria Poca^2,5^, Cristina Gely^2^, Juan Camilo Nieto^3^, Xavier Torras^2,5^, Eva Román^2,4,5^, David Campos^1^, Guillaume Sarrabayrouse^1^, Silvia Vidal^3^, Francisco Guarner^1,5^, German Soriano^2,5^, Chaysavanh Manichanh^1,5^, Carlos Guarner^2,5^

^1^Digestive System Research Unit, Vall d’Hebron Research Institute, Passeig Vall d’Hebron 119-129, Barcelona 08035, Spain

^2^Department of Gastroenterology, ^3^Department of Immunology, Institut de Recerca-IIB Sant Pau, and ^4^Escola Universitària d’Infermeria EUI-Sant Pau, Hospital de la Santa Creu i Sant Pau, Barcelona, Spain

^5^Centro de Investigaciòn Biomédica en Red en el Área temática de Enfermedades Hepáticas y Digestivas, CIBERehd, Instituto de Salud Carlos III, Madrid, Spain

Correspondence: Chaysavanh Manichanh and German Soriano, co-corresponding authors

Dr. Chaysavanh Manichanh,

Lab 17, Edificio Mediterranea, Vall d’Hebron Research institute, Passeig Vall d’Hebron 119-129, 08035 Barcelona, Spain

Email: cmanicha@gmail.com

Dr. German Soriano

Department of Gastroenterology, Hospital de la Santa Creu i Sant Pau

Mas Casanovas, 90, 08041 Barcelona Spain

E-Mail: gsoriano@santpau.cat

**Supplementary Figures**


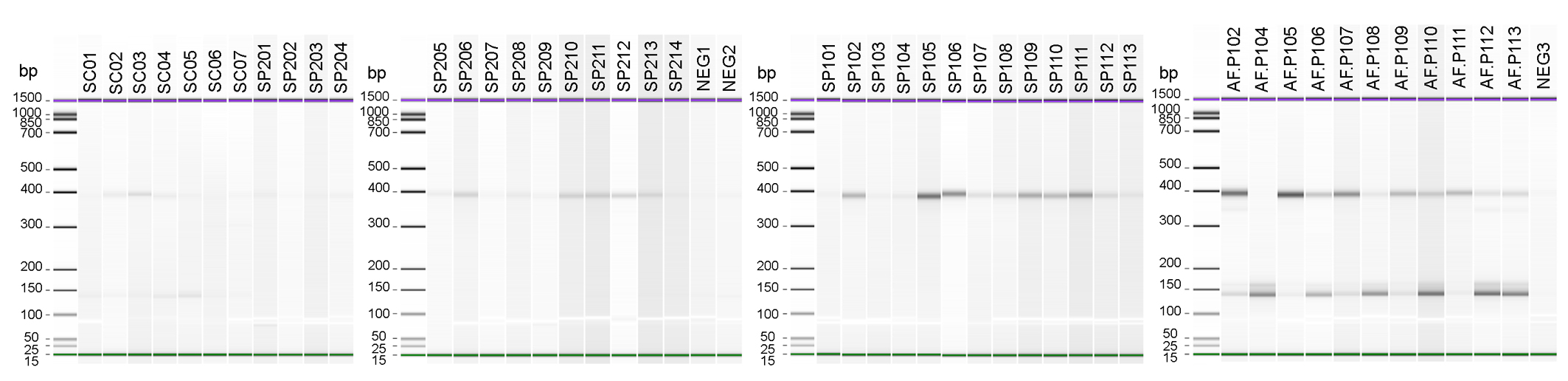


**Supplementary Fig. 1. Analysis of the PCR amplifications of the V4 region of the 16S rRNA gene on an electrophoretic gel.** The products of the PCR amplification were loaded on an Agilent 2100 Bioanalyzer chip using the Agilent 1000 kit. The presence of the 16S gene amplicon can be detected at around 400 bp. The presence of DNA bands at around 150 bp can be identified as the primer dimers that are removed during sequence analysis.


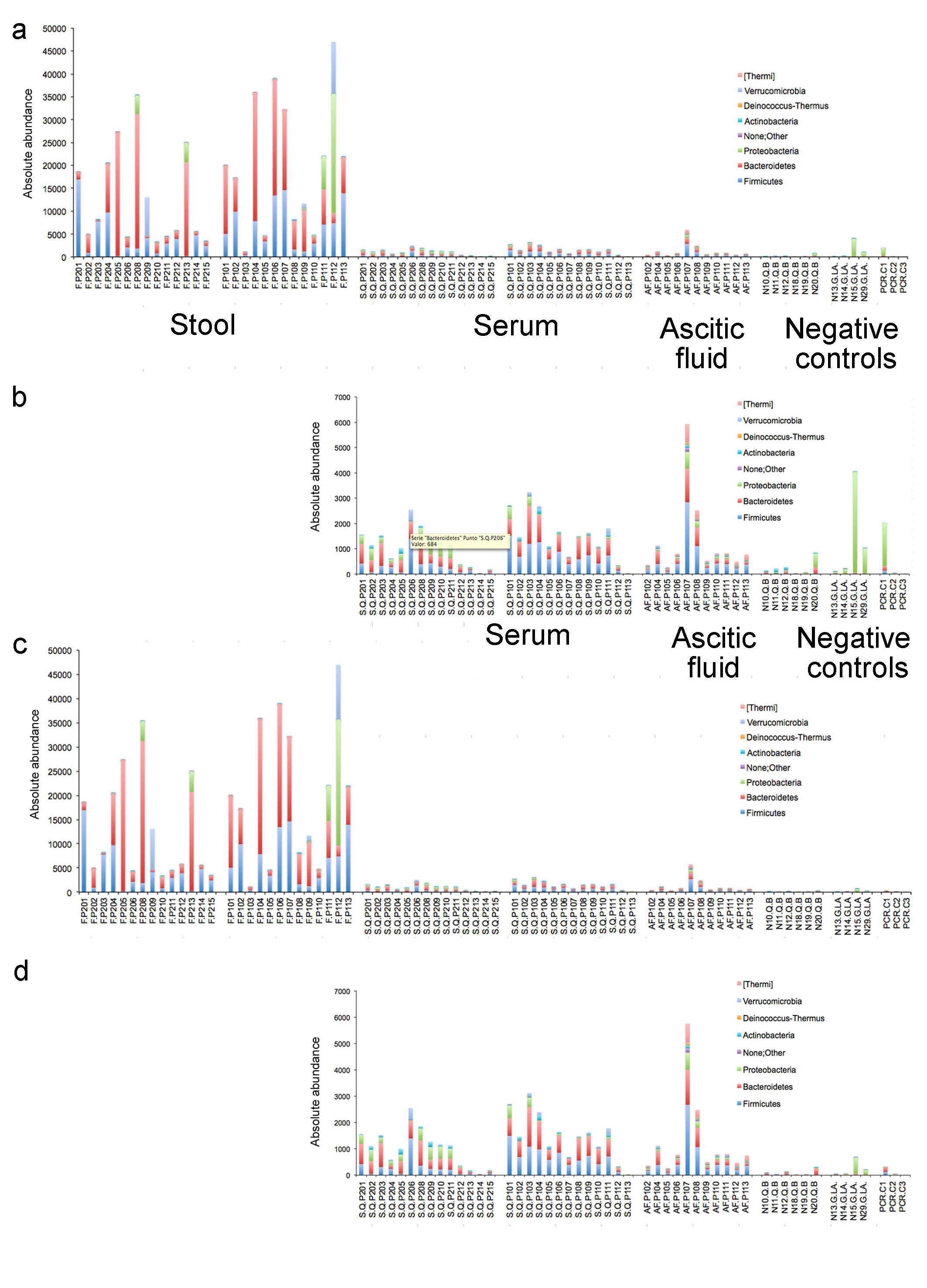


**Supplementary Fig. 2. Taxonomic profiling at the phylum level.** (**a,b**) before filtering out contaminant sequences. (**c,d**) and after filtering out contaminant sequences. F = fecal sample; S = serum; AF = ascitic fluid; P = patient. Abundance of the phyla is given as absolute values.


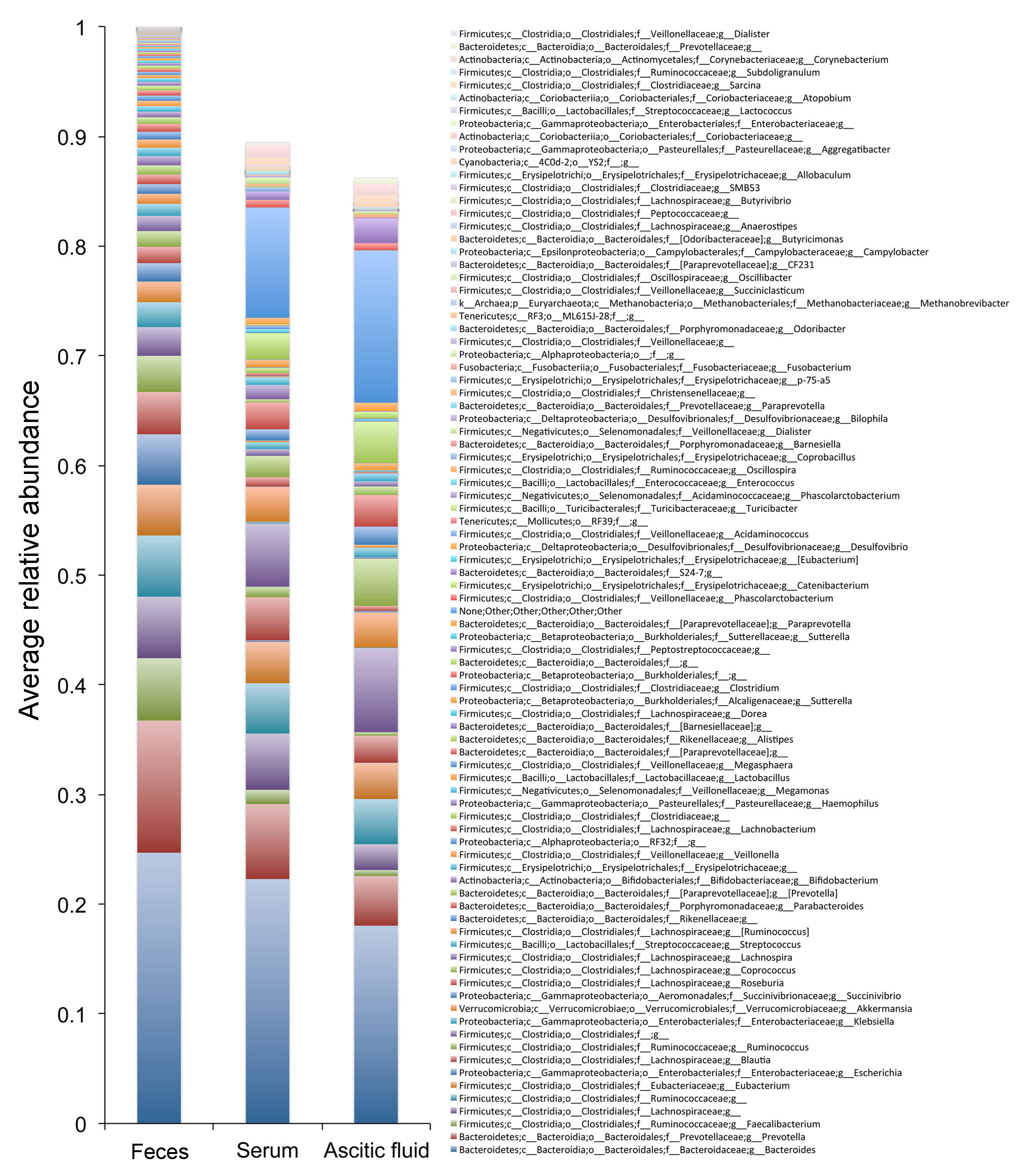


**Supplementary Fig.3. Taxonomic profiling of the 3 types of samples**: feces, serum and ascitic fluid.


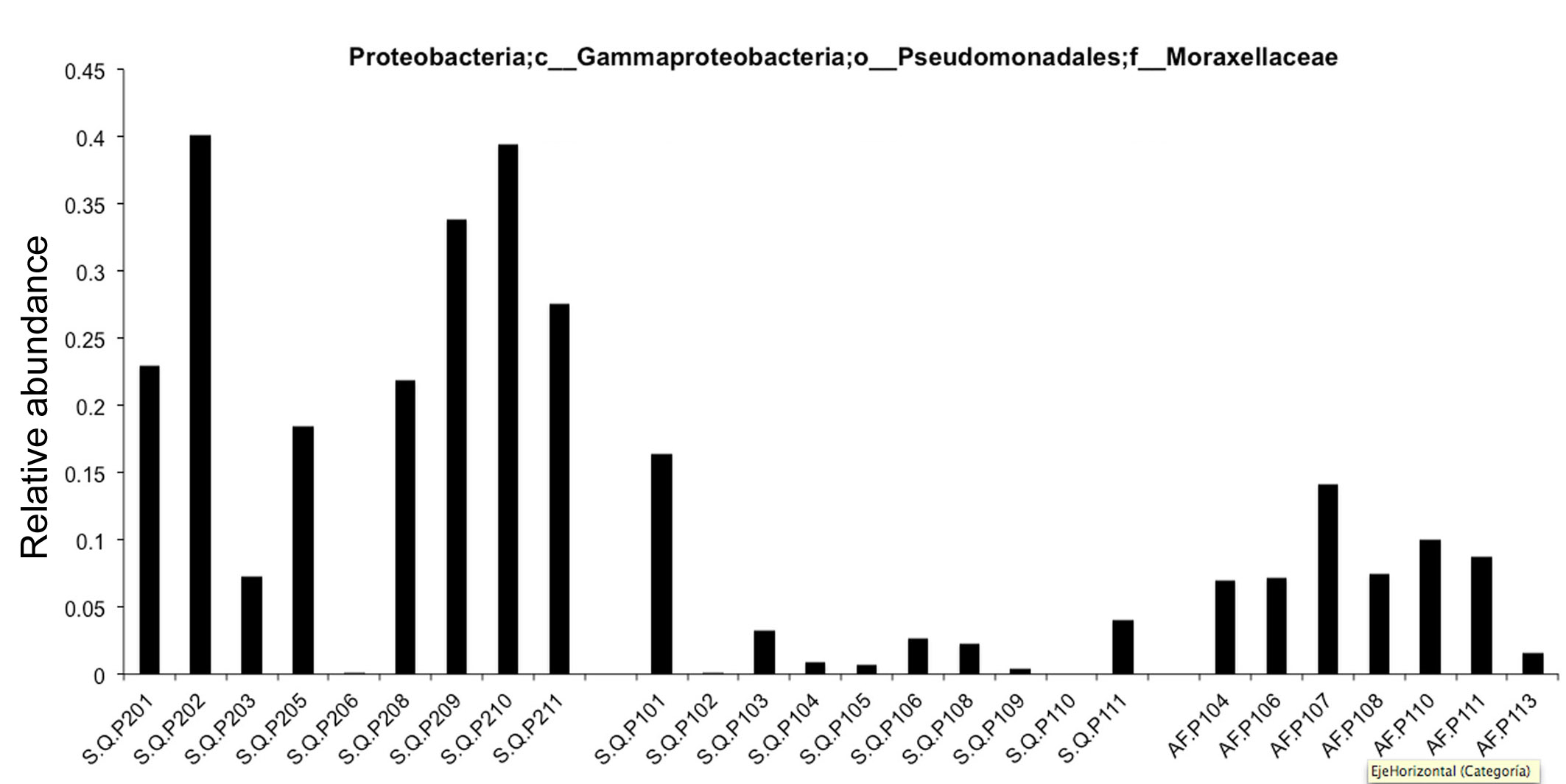


**Supplementary Fig. 4. Higher relative abundance of Moraxellaceae in serum of patients without ascites and ascitic fluid samples than in serum of patients with ascites.** Analyses were performed on 16S rRNA V4 region data, rarefied to a depth of 700 reads per sample.


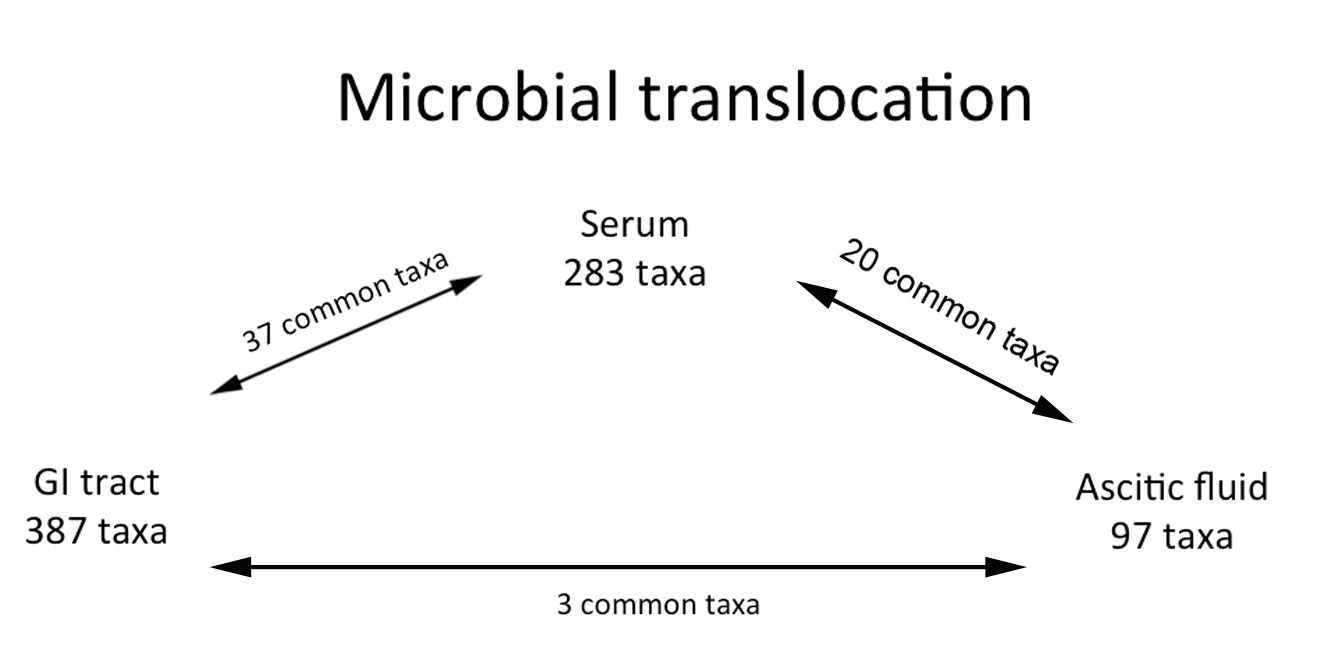


**Supplementary Fig. 5. Microbial translocation.** The average number of taxa is indicated under each sample type. The average number of taxa common to two sample types is indicated between sample types.

**Supplementary Table 1.** **Clinical and analytical characteristics of patients in each group (with and without ascites).** Results expressed as mean ± SEM or number of patients (%).

|  | **Ascites**  **(n = 13)** | **No ascites**  **(n = 14)** | **p** |
| --- | --- | --- | --- |
| Age (years) | 68.9±3.0 | 67.3±2.3 | 0.69 |
| Gender (male/female) | 9 (69%)/4 (31%) | 10 (71%)/4 (28%) | 1.00 |
| Body mass index (kg/m^2^) | 28.0±0.9 | 28.5±0.6 | 0.49 |
| Diabetes | 6 (46%) | 4 (27%) | 0.44 |
| Child-Pugh A/B/C | 3 (23%)/9 (69%)/1 (8%) | 9 (64%)/4 (28%)/ 1(7%) | 0.08 |
| Child-Pugh score | 7.7±0.4 | 6.1±0.4 | **0.008** |
| MELD score | 11.6±0.9 | 9.5±1.0 | 0.08 |
| Etiology (alcohol/virus/alcohol+  virus/other) | 7 (54%)/3 (23%)/1 (8%)  2 (15%) | 9 (64%)/2 (14%)/ 0 (0%)/ 3 (21%) | 0.49 |
| Previous decompensation of cirrhosis | 13 (100%) | 9 (64%) | **0.04** |
| Previous ascites | 13 (100%) | 9 (64%) | **0.04** |
| Previous encephalopathy | 1 (8%) | 4 (29%) | 0.32 |
| Previous variceal bleeding | 2 (15%) | 0 (0%) | 0.22 |
| Beta-blockers | 3 (23%) | 6 (43%) | 0.42 |
| Diuretics | 9 (69%) | 7 (50%) | 0.44 |
| Proton pump inhibitors | 3 (23%) | 7 (50%) | 0.23 |

**Supplementary Tables**

**Supplementary Table 2. Rarefaction depths for sequences analyzed for each sample type.**

|  | **Total** | **Mean** | **Minimum** | **Maximum** | **Rarefaction depth** |
| --- | --- | --- | --- | --- | --- |
| **Feces** | 2,008,492 | 45,647 | 19,930 | 131,334 | 19,930 |
| **Serum** | 117,322 | 4,345 | 204 | 8,497 | 1,000 |
| **Ascitic fluid** | 24,359 | 2,214 | 381 | 8,930 | 1,000 |
